# LncRNA *CACF* promotes autophagy and cardiac recovery after myocardial infarction by targeting *ATG7*

**DOI:** 10.1016/j.isci.2026.115889

**Published:** 2026-04-28

**Authors:** Jinghao Yang, Guofeng Bai, Weili Liao, Qingyang Zhao, Nian Li, Xiaofeng Zhou, Yingting He, Jingyu Zhou, Hongyan Quan, Chennan Lin, Yixuan Guo, Liuhong Zhang, Enyuan Huang, Linfang Yang, Jianghua Zeng, Hao Zhang, Xiaolong Yuan, Xilong Wang

**Affiliations:** 1 Guangdong Provincial Biotechnology Research Institute (Guangdong Provincial Laboratory Animals Monitoring Center), Guangzhou 510663, China; 2 National Engineering Research Center for Breeding Swine Industry, State Key Laboratory of Swine and Poultry Breeding Industry, Guangdong Provincial Key Laboratory of Agro-Animal Genomics and Molecular Breeding, College of Animal Science, South China Agricultural University, Guangzhou, Guangdong 510642, China; 3 Guangxi Key Laboratory of Animal Breeding and Disease Control, College of Animal Science and Technology, Guangxi University, Nanning 530004, China; 4 College of Veterinary Medicine, South China Agricultural University, Guangzhou, Guangdong 510642, China; 5 Guangdong Yihao Local Pig Research Institute Co., Zhanjiang, Guangdong 524000, China

**Keywords:** biological sciences, cell biology, genetics

## Abstract

Myocardial infarction (MI) is a severe global health issue with high morbidity and mortality. Recent studies highlight the critical role of lncRNAs in recovery following MI but the molecular mechanisms remain largely unclear. In this study, we identified a novel lncRNA, cardiac autophagy contributory factor (CACF), in an MI model of miniature pigs. Our results demonstrate that CACF promotes angiogenesis in vascular cells by modulating autophagy through miR-520b-3p and miR-20a-5p. CACF binds negatively to miR-520b-3p and miR-20a-5p, upregulating ATG7 to enhance autophagy and cardiac recovery. This mechanism promotes cell proliferation and inhibits apoptosis in vascular cells. Notably, miR-20a-5p increases cell proliferation and reduces apoptosis, while miR-520b-3p inhibits proliferation and promotes apoptosis. Both miRNAs suppress autophagy. The interplay among CACF, miR-520b-3p, miR-20a-5p, and ATG7 reveals a complex regulatory network that enhances autophagy and cardiac repair after MI. These findings provide potential insights for therapeutic strategies in MI.

## Highlights

• Identified a lncRNA CACF involved in post-MI cardiac repair
• CACF promotes angiogenesis via autophagy modulation in vascular cells
• CACF enhances ATG7 expression by targeting miR-520b-3p and miR-20a-5p
• CACF-miRNA-ATG7 axis reveals a novel autophagy-related repair mechanism

## Graphical abstract

**Figure:**
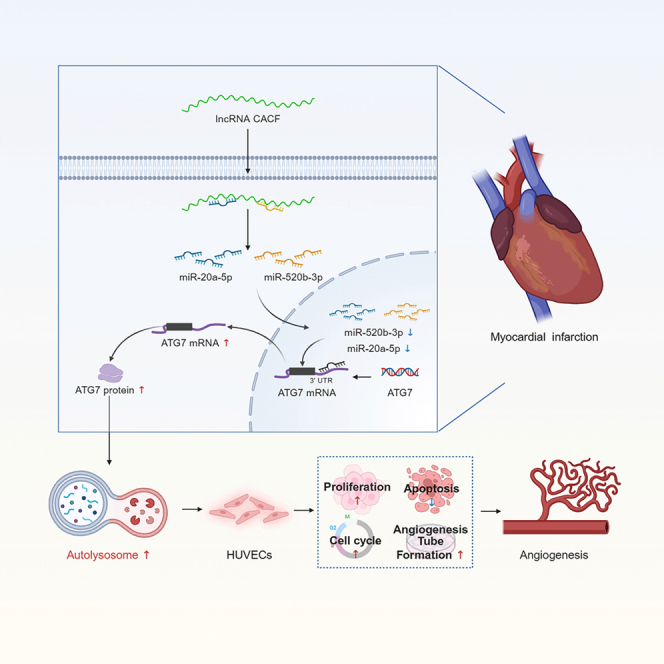

## Introduction

Annually, myocardial infarction (MI) impacts approximately 30–40 million individuals worldwide.^1^ Recent trends indicate a younger demographic becoming susceptible to MI.^2^ However, the complex etiology and pathophysiology of MI pose challenges to the development of effective preventative and therapeutic strategies, which remain both limited and costly. During MI, hypoxia-induced apoptosis of vascular endothelial cells diminishes the density of capillary, exacerbating left ventricular dysfunction and leading to heart failure.^3^^,^^4^^,^^5^ Therefore, rapid restoration of blood supply and angiogenesis in infarcted areas are crucial for cardiac recovery after MI.^6^^,^^7^ Physiologically, autophagy helps to maintain cellular homeostasis, whereas MI often induces severe autophagic dysfunction, impairing its ability to participate in post-MI regulation.^8^ After MI, autophagy protects the heart by promoting the accumulation of normal proteins and inhibiting the cardiac oxidative injury.^9^ It has been reported that the increased level of autophagy may represent a beneficial strategy for cardiac protection.^10^

The family of autophagy-related genes (ATGs) participates in the initiation, elongation, maturation, fusion, and degradation of autophagy, regulating the occurrence and degradation of various autophagy-mediated pathways.^10^ Previous study has shown that autophagy in vascular endothelial cells activates angiogenesis after MI.^11^ The disruption of autophagy-related gene 6 (*ATG6*) transcription markedly reduces the expression of vascular endothelial growth factor A (VEGFA) and leads to angiogenesis in a mouse MI model.^12^ Activation of autophagy-related gene 5 (*ATG5*) in the heart and autophagy-related gene 7 (*ATG7*) in the pancreas effectively alleviates the susceptibility to acute MI in aged rats.^13^ In recent years, non-coding RNAs have increasingly been shown to participate in the regulation of the ATG family, and they function as molecular sponges and competitively bind to miRNAs, thereby regulating the transcription of mRNAs.^14^^,^^15^ In cardiac diseases, many specific long noncoding RNAs (lncRNAs) have been demonstrated to regulate cardiac repair mechanisms. *LncAPF* enhances autophagy in cardiomyocytes and improves cardiac function in a mouse MI model through its interaction with miR-188-3p, upregulating the expression of *ATG7*.^16^ The upregulation of *LncRNA H19* in mouse myocardium significantly activate *ATG7*-mediated autophagy, thereby significantly reducing infarct size.^17^ These findings suggest that lncRNAs may regulate *ATGs* to promote the cardiac recovery. However, the regulatory pathways involved in this process still need further exploration and refinement.

In this study, we identified a lncRNA named cardiac autophagy contributory factor (CACF), which plays a critical role in the regulation of autophagy. CACF competitively binds to miR-520b-3p and miR-20a-5p, enhancing the expression of *ATG7* and promoting the cardiac recovery. This regulatory mechanism underscores the potential of targeting lncRNA-mediated autophagy pathways as a therapeutic strategy for MI recovery.

## Results

### Identification and functional characterization of differentially expressed lncRNAs in infarcted cardiac tissues

**Figure 1:**
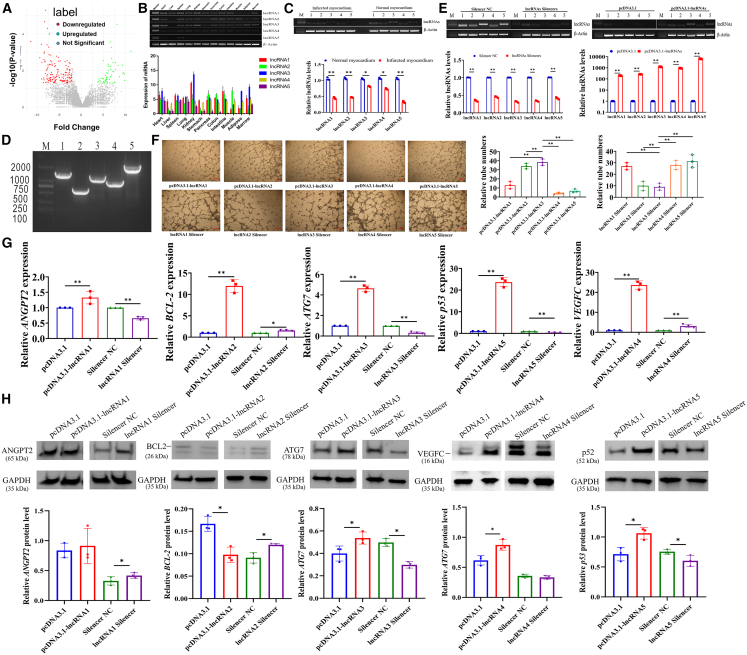
Identification and functional characterization of differentially expressed lncRNAs in infarcted cardiac tissues (A) Expressions of lncRNAs in MI tissues, compared to normal tissues in pigs. (B) Expression profiles of five lncRNAs across different tissues. (C) Expressions of five lncRNAs in the infarcted myocardium and normal myocardium. (D) Expressions of five lncRNAs in the human genome. (E) Changes in the expressions of five lncRNAs in vascular cells treated with their overexpression plasmids and smart silencers, respectively. (F) Influences of five lncRNAs on the number of tubes formed by vascular cells. Effects of five lncRNAs on the mRNA (G) and protein (H) levels of their target genes. Data are shown as mean ± SEM; ∗*p* < 0.05, ∗∗*p* < 0.01; unpaired two-tailed Student’s *t* test. Scale bars, 200 μm in (F); *n* = 3 biological replicates per group.

In our study, we performed high-throughput RNA sequencing on infarcted tissues from MI miniature pigs and corresponding tissues from normal miniature pigs. The analysis identified a total of 870 differentially expressed lncRNAs (Figure 1A). Previous studies have reported B cell lymphoma 2 gene (*BCL2*),^18^^,^^19^^,^^20^ angiopoietin-2 (*ANGPT2*),^21^^,^^22^^,^^23^ *ATG7*,^16^^,^^24^^,^^25^ vascular endothelial growth factor C (VEGFC),^26^^,^^27^^,^^28^ and tumor protein 53 (*p53*)^29^^,^^30^^,^^31^ might regulate the development of MI, and we tried to identify the highly differentially expressed lncRNAs that might regulate these important genes in the MI group. The nearest genomic distance of these functional genes revealed five differentially expressed lncRNAs: XLOC_110286 (lncRNA1), XLOC_025806 (lncRNA2), XLOC_075168 (lncRNA3), XLOC_102237 (lncRNA4), and XLOC_057528 (lncRNA5) (Table S2). We found that lncRNA3 was predominantly expressed in cardiac tissue compared to other lncRNAs (Figure 1B), and all five lncRNAs exhibited significantly lower expression in the infarcted myocardium, compared to the normal myocardium (Figure 1C). Moreover, we discovered that these 5 lncRNAs were also expressed in the human genome, with total lengths of 1,555, 819, 1,358, 1,018, and 2,085 nt for lncRNA1 to lncRNA5, respectively (Figure 1D).

We further overexpressed and knocked down these lncRNAs (Figure 1E). We found that overexpression of lncRNA3 enhanced the tube formation ability of vascular cells the most among the five lncRNAs significantly, and inhibited the expression of lncRNA3 decreased the tube formation ability of vascular cells the most (Figure 1F). Meanwhile, lncRNA3 and lncRNA5 significantly enhanced the proliferation of vascular cells and inhibited apoptosis (Figures S1A and S1B). Moreover, lncRNA3 notably promoted the levels of both mRNA and protein of *ATG7* (*p* < 0.01) (Figures 1G and 1H), but other lncRNAs exhibited the inconsistent trends in their effects on the mRNAs and proteins expression levels of their target genes (Figures 1G and 1H). Based on the effects of lncRNA3 on the proliferation, apoptosis, and tube formation of vascular cells, we speculated that lncRNA3 might be a potential factor influencing MI by regulating autophagy. We named lncRNA3 as CACF.

### CACF promoted the proliferation and cell cycle progression and inhibited the apoptosis of vascular cells

**Figure 2:**
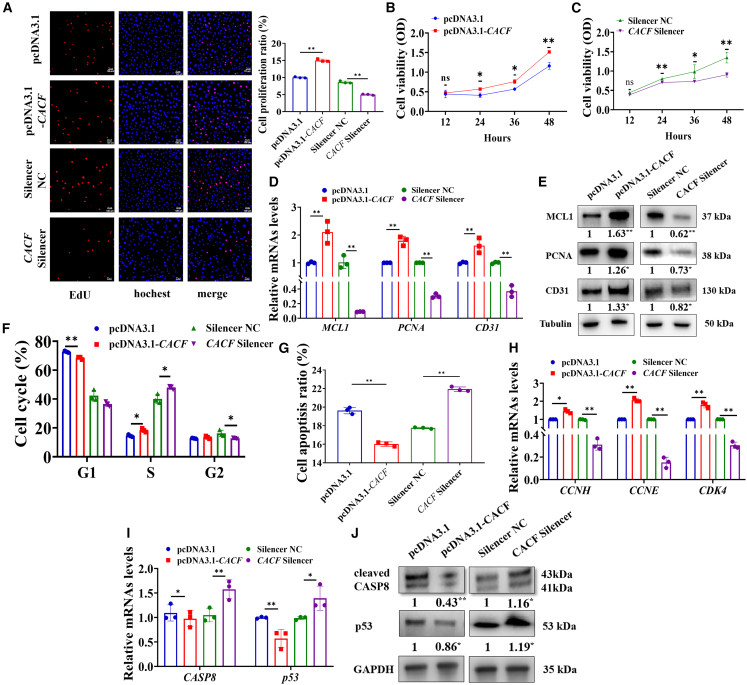
CACF promoted proliferation and cell cycle progression and inhibited the apoptosis of vascular cells (A) Effect of CACF on the cellular proliferation. Effects of pcDNA3.1-*CACF* (B) and CACF silencer (C) on the viabilities of vascular cells. Effects of CACF on the mRNA (D) and protein (E) levels of proliferation-related genes. Effects of CACF on the cell cycle (F) and apoptosis (G). Effects of CACF on the levels of cell cycle-related genes (H), apoptosis-related genes (I), and apoptosis-related proteins (J). Data are shown as mean ± SEM; ∗*p* < 0.05, ∗∗*p* < 0.01; unpaired two-tailed Student’s *t* test. Scale bars, 100 μm in (A); *n* = 3 biological replicates per group.

**Figure 3:**
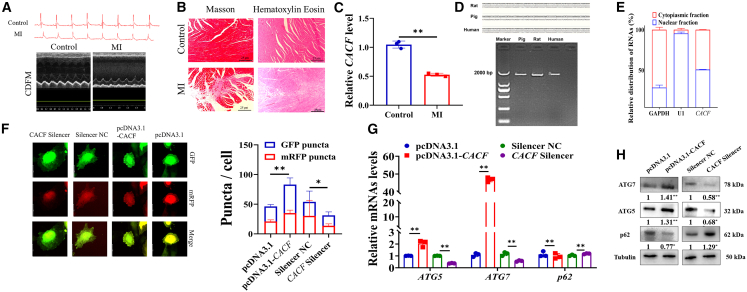
CACF might play a crucial role in MI by promoting autophagy (A) The electrocardiogram of rats after LAD. (B) Changes of histological and pathological of rat heart tissues after MI. (C) The expression of CACF in heart after MI of rats. (D) The expressions of CACF in the genomes of humans, rats, and pigs. (E) The distribution of CACF in the cytoplasm and nucleus in vascular cells. *U1* small nuclear RNA was used as an endogenous nuclear reference, whereas *GAPDH* was used as a cytoplasmic reference. (F) Effect of CACF on the formation of autophagy flux in vascular cells. Effect of CACF on the levels of autophagy-related genes (G) and autophagy-related proteins (H). Data are shown as mean ± SEM; ∗*p* < 0.05, ∗∗*p* < 0.01; unpaired two-tailed Student’s *t* test. Scale bars: 25 μm in (B) and 10 μm in (F); *n* = 3 biological replicates per group.

We found that pcDNA3.1-CACF significantly promoted the proliferation, while CACF silencer significantly inhibited the proliferation of vascular cells (Figure 2A). Similarly, the viabilities of vascular cells were also enhanced by pcDNA3.1-CACF (Figure 2B), while CACF silencer significantly diminished the viabilities of vascular cells (Figure 2C). Additionally, quantitative reverse-transcription PCR (RT-qPCR) analysis indicated a notable upregulation in the mRNA levels of key proliferation-associated genes, including *MCL1* (*p* < 0.01), *PCNA* (*p* < 0.01), and *CD31* (*p* < 0.01) (Figure 2D). Western blot analysis demonstrated significantly elevated levels of proliferation-related proteins, including MCL1 (*p* < 0.01), PCNA (*p* < 0.05), and CD31 (*p* < 0.05) (Figure 2E). Furthermore, CACF significantly accelerated the progression of cell cycle (Figure 2F) and significantly inhibited apoptosis in vascular cells (Figure 2G). RT-qPCR analysis further demonstrated a significant upregulation in the mRNA levels of cell cycle-promoting genes, including *CCNH* (*p* < 0.05), cyclin E (CCNE) (*p* < 0.01), and *CDK4* (*p* < 0.01) (Figure 2H), and there was a significant downregulation in the mRNA levels of apoptosis-inducing genes, including, cysteinyl aspartate specific proteinase 8 (CASP8) (*p* < 0.05) and *p53* (*p* < 0.01) (Figure 2I). Western blot analysis further confirmed that CACF significantly reduced the levels of apoptosis-inducing proteins, including cleaved *CASP8* (*p* < 0.01) and *p53* (*p* < 0.05) (Figure 3J).

### CACF might play a crucial role in MI by promoting autophagy

Based on results of Figure 1, CACF was supposed to regulate autophagy in MI. To assess the role of CACF in the progression of MI, we established an MI model in rats. Electrocardiographic monitoring showed persistent ST-segment elevation, and doppler echocardiography revealed slight fluctuations in the anterior wall of the left ventricle in rats after MI (Figure 3A). We found that the myocardial collagen fibers were increased and disorganized in the MI group (Figure 3B), as well as inflammatory infiltration in the extracellular matrix (Figure 3B), compared to the control group. These results confirmed the successful establishment of the rat MI model. Moreover, the significant decrease in the expression of CACF in the MI group might suggest a pivotal role for CACF in MI (Figure 3C).

The expression of CACF was detected across different species, including humans, rats, and pigs, and demonstrating its conservatism (Figure 3D). Furthermore, we found that CACF was expressed in both the nucleus and cytoplasm (Figure 3E). The results of GFP-mRFP-LC3 showed that CACF increased the formation of autophagic flux in vascular cells (Figure 3F). RT-qPCR analysis revealed that CACF significantly upregulated the mRNA levels of *ATGs* including *ATG7* (*p* < 0.01) and *ATG5* (*p* < 0.01), and downregulated *p62* (*p* < 0.01) (Figure 3G). Consistent, the protein levels of ATG7 (*p* < 0.01) and ATG5 (*p* < 0.01) were significantly increased, whereas *p62* (*p* < 0.05) level was decreased (Figure 3H), and these results were consistent with Figures 1G and 1H.

### CACF promoted autophagy by competitively binding to miR-520b-3p and miR-20a-5p to promote the expression of *ATG*7

**Figure 4:**
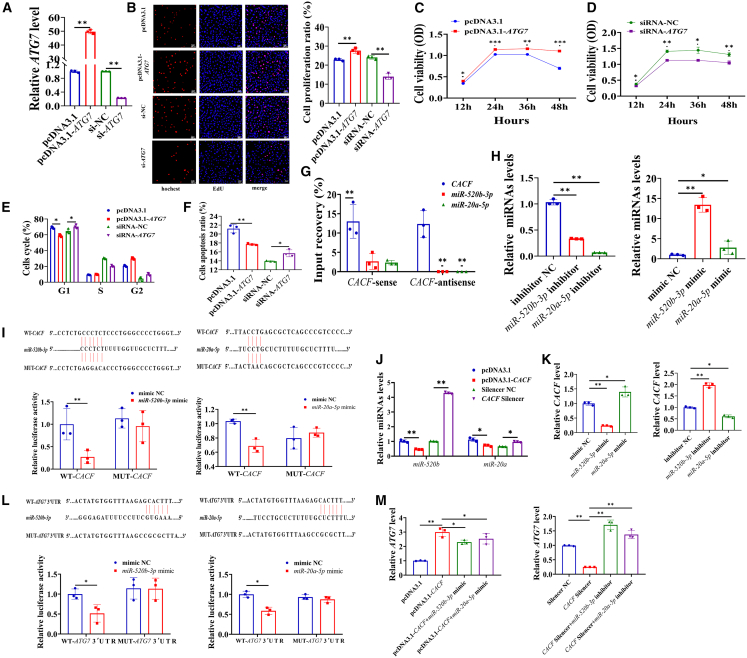
CACF promoted autophagy by competitively binding to miR-520b-3p and miR-20a-5p to promote the expression of *ATG*7 (A) The mRNA level of *ATG7* in vascular cells treated with pcDNA3.1, pcDNA3.1-*ATG7*, si-NC, and si-*ATG7*. (B) Effect of *ATG7* on the proliferation of vascular cells. (C) Effect of pcDNA3.1-*ATG7* on the viability of vascular cells. (D) Effect of si-*ATG7* on the viability of vascular cells. Effect of ATG7 on the cell cycle (E) and apoptosis (F). (G) The levels of miR-520b-3p and miR-20a-5p in CACF-sense and CACF-antisense pull-down complexes. (H) The miRNA levels of miR-520b-3p and miR-20a-5p in vascular cells treated with mimics and silencers, separately. (I) The fluorescence activity of CACF after co-transfection of wild-type/mutant CACF, miR-520b-3p mimic/mimic NC and miR-20a-5p mimic/mimic NC. (J) Effect of CACF on the miRNA levels of miR-520b-3p and miR-20a-5p in vascular cells. (K) Effects of miR-520b-3p and miR-20a-5p on the level of CACF in vascular cells. (L) The fluorescence activity of *ATG7* after co-transfection of the wild-type/mutant *ATG7* 3′ UTR, miR-520b-3p mimic/mimic NC and miR-20a-5p mimic/mimic NC. (M) Effects of miR-520b-3p and miR-20a-5p on the level of CACF in vascular cells. Data are shown as mean ± SEM; ∗*p* < 0.05, ∗∗*p* < 0.01; unpaired two-tailed Student’s *t* test. Scale bars, 100 μm in (B); *n* = 3 biological replicates per group.

We constructed the overexpression plasmids and small interfering RNA (siRNA) of *ATG7*, and the mRNA level of *ATG7* was significantly increased at a concentration of 100 ng/mL of pcDNA3.1-*ATG7*, whereas significantly decreased at 50 nM of siRNA-*ATG7* (Figure 4A). *ATG7* significantly promoted vascular cell proliferation (Figure 4B). Similarly, the vascular cells viability was significantly enhanced by pcDNA3.1-*ATG7* (Figure 4C), while siRNA-*ATG7* significantly inhibited vascular cell viability (Figure 4D). Moreover, ATG7 significantly accelerated the progression of cell cycle (Figure 4E), and inhibited cell apoptosis (Figure 4F).

We discovered through probes that CACF did not directly regulate the expression of ATG7 through binding. Previous studies demonstrated that lncRNAs exerted regulatory effects by binding to miRNAs. We screened the miRbase database (https://www.mirbase.org) and identified potential binding sites for miR-520b-3p and miR-20a-5p with both CACF and *ATG7*. Pull-down assays confirmed specific interactions between CACF and these miRNAs, with successful enrichment of miR-520b-3p and miR-20a-5p by using the CACF sense probe, whereas the antisense probe showed no interaction (Figure 4G).

To further probe these interactions, we synthesized mimics and inhibitors for miR-520b-3p and miR-20a-5p, observing the significantly effects at concentrations of 50 nm for mimics and 100 nm for inhibitors (Figure 4H). The luciferase activity of wild-type (WT)-CACF decreased after treatment with both miR-520b-3p and miR-20a-5p mimic (Figure 4I). These findings were supported by RT-qPCR, and the results showed pcDNA3.1-CACF reduced the levels of miR-520b-3p and miR-20a-5p, and CACF silencer increased the levels of miR-520b-3p and miR-20a-5p (Figure 4J). Additionally, miR-520b-3p increased the level of CACF but miR-20a-5p reduced the level of CACF (Figure 4K). *ATG7* 3′UTR to assess its targeting interaction with the miRNAs, and both miR-520b-3p and miR-20a-5p mimic decreased the activity of WT-*ATG7* 3′ UTR (Figure 4L). RT-qPCR showed that pcDNA3.1-CACF significantly increased the mRNA level of *ATG7*, and this effect was reversed by the miR-520b-3p mimic and miR-20a-5p mimic (Figure 4M). While CACF silencer significantly decreased the mRNA level of ATG7, and this inhibition was reversed by the miR-520b-3p inhibitor and miR-20a-5p inhibitor (Figure 4M).

### MiR-520b-3p inhibited proliferation and the cell cycle, and promoted the apoptosis of vascular cells, while miR-20a-5p showed the opposite effect

**Figure 5:**
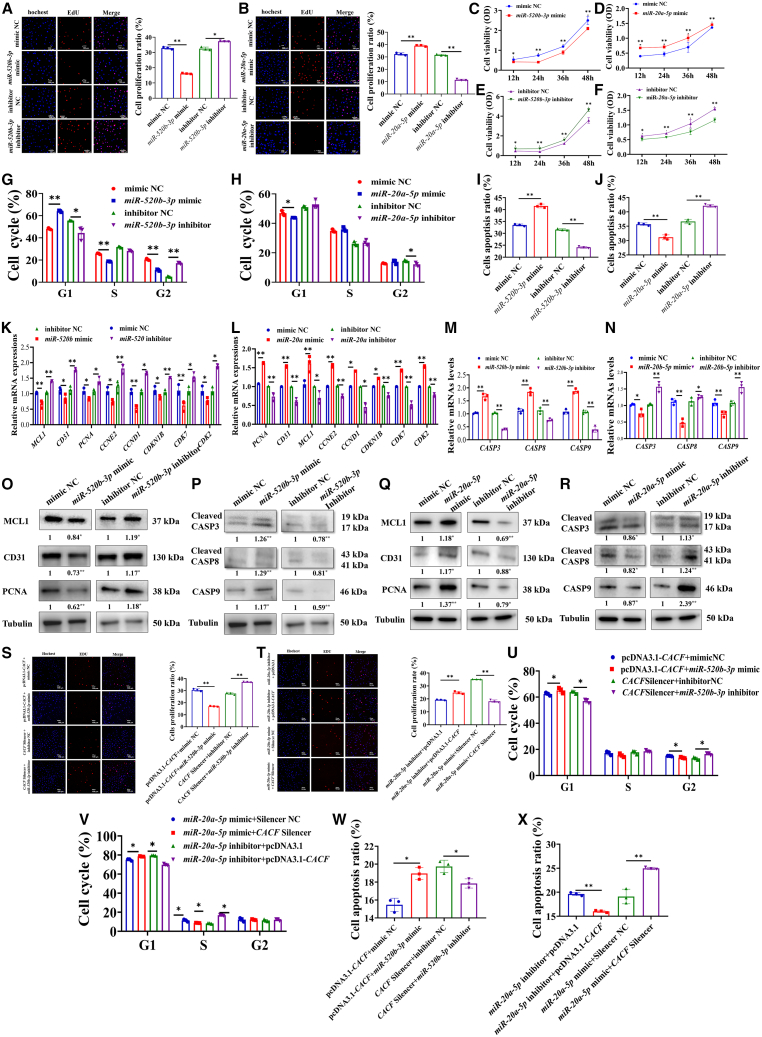
MiR-520b-3p inhibited proliferation and the cell cycle and promoted the apoptosis of vascular cells, while miR-20a-5p had the opposite effect Effects of miR-520b-3p (A) and miR-20a-5p (B) on the proliferation of vascular cells. Effects of miR-520b-3p mimic (C) and inhibitor (D) on the viability of vascular cells. Effects of miR-20b-5p mimic (E) and inhibitor (F) on the viability of vascular cells. Effects of miR-520b-3p (G) and miR-20a-5p (H) on the progression of cell cycle of vascular cells. Effects of miR-520b-3p (I) and miR-20a-5p (J) on the apoptosis of vascular cells. Effect of miR-520b-3p on the expression levels of proliferation marker genes and cell cycle marker genes (K). Effect of miR-20a-5p on the expression levels of proliferation marker genes and cell cycle marker genes (L). Effect of miR-520b-3p (M) and miR-20a-5p (N) on the expression levels of apoptosis marker genes. Effect of miR-520b-3p on the levels of proliferation marker proteins (O) and apoptosis marker proteins (P), and the detected proteins were derived from the same membrane or obtained through stripping and reprobing. Effect of miR-20a-5p on the levels of proliferation marker proteins (Q) and apoptosis marker proteins (R), and the detected proteins were derived from the same membrane or obtained through stripping and reprobing. (S) Effect of miR-520b-3p on the proliferation of vascular cells treated with pcDNA3.1-CACF and CACF silencer (scale bars, 100 μm). (T) Effect of CACF on the proliferation of vascular cells treated with miR-20a-5p inhibitor and miR-20a-5p mimic. (U) Effect of miR-520b-3p on the cell cycle of vascular cells treated with pcDNA3.1-CACF and CACF silencer. (V) Effect of CACF on the cell cycle of vascular cells treated with miR-20a-5p inhibitor and miR-20a-5p mimic. (W) Effect of miR-520b-3p on the apoptosis of vascular cells treated with pcDNA3.1-CACF and CACF silencer. (X) Effect of CACF on the apoptosis of vascular cells treated with miR-20a-5p inhibitor and miR-20a-5p mimic. Data are shown as mean ± SEM; ∗*p* < 0.05, ∗∗*p* < 0.01; unpaired two-tailed Student’s *t* test. Scale bars, 100 μm in (A, B, S, and T); *n* = 3 biological replicates per group.

We found that miR-520b-3p markedly inhibited the proliferation of vascular cells (Figure 5A) but miR-20a-5p significantly promoted the proliferation of vascular cells (Figure 5B). Similarly, miR-520b-3p mimic significantly inhibited the viability of vascular cells (Figure 5C), and miR-520b-3p inhibitor enhanced the viability of vascular cells (Figure 5E). MiR-20a-5p mimic significantly enhanced the viability of vascular cells (Figure 5D), and miR-20a-5p inhibitor reduced the viability of vascular cells (Figure 5F). Flow cytometry analyses showed that miR-520b-3p mimic inhibited the progression of cell cycle (Figure 5G), and increased apoptosis rates (Figure 5I), while miR-20a-5p had opposing effects (Figures 5H and 5J).

Results of RT-qPCR indicated miR-520b-3p significantly downregulated several proliferation marker genes and cell cycle marker genes by miR-520b-3p, e.g., *MCL1* (*p* < 0.01), *CD31* (*p* < 0.05), *PCNA* (*p* < 0.05), *CCNE2* (*p* < 0.05), *CCND1* (*p* < 0.01), *CDKN1B* (*p* < 0.05), *CDK7* (*p* < 0.01) and *CDK2* (*p* < 0.05) (Figure 5K). In contrast, *miR-20a-5p* significantly upregulated the expression levels of proliferation marker genes and cell cycle marker genes, e.g., *MCL1* (*p* < 0.01), *CD31* (*p* < 0.01), *PCNA* (*p* < 0.01), *CCNE2* (*p* < 0.01), *CCND1* (*p* < 0.01), *CDKN1B* (*p* < 0.01), *CDK7* (*p* < 0.01) and *CDK2* (*p* < 0.01) (Figure 5L). Besides, *miR-520b-3p* also significantly increased the mRNA levels of the apoptosis marker genes, e.g., *CASP3* (*p* < 0.01), *CASP8*(*p* < 0.01) and *CASP9* (*p* < 0.01), while *miR-20a-5p* had the opposite effects (Figures 5M and 5N). Consistently, the results of western blot analysis showed that *miR-520b-3p* reduced the levels of proliferation marker proteins, e.g., MCL1 (*p* < 0.05), PCNA (*p* < 0.01) and CD31 (*p* < 0.01) (Figure 5O), and increased the levels of apoptosis marker proteins, e.g., CASP3 (*p* < 0.01), CASP8 (*p* < 0.01), and CASP9 (*p* < 0.05) (Figure 5P). Conversely, miR-20a-5p prominently elevated the levels of MCL1 (*p* < 0.05), PCNA (*p* < 0.01) and CD31 (*p* < 0.05) (Figure 5Q), and reduced the levels of CASP3 (*p* < 0.05), CASP8 (*p* < 0.05) and CASP9 (*p* < 0.05) (Figure 5R). Indicating both miR-520b-3p and miR-20a-5p had regulatory effects in cell proliferation and apoptosis.

Moreover, the promotion of pcDNA3.1-CACF on proliferation was reversed by miR-520b-3p mimic, and the inhibition of CACF silencer on proliferation was reversed by miR-520b-3p inhibitor (Figure 5S). The inhibition of miR-20a-5p inhibitor on proliferation was reversed by pcDNA3.1-CACF, and the promotion of miR-20a-5p mimic on proliferation in vascular cells was reversed by CACF silencer (Figure 5T). Flow cytometry revealed that the acceleration of pcDNA3.1-CACF on the progression of cell cycle was reversed by miR-520b-3p mimic, the deceleration of CACF silencer on the progression of cell cycle was reversed by miR-520b-3p inhibitor (Figure 5U). The acceleration of the progression of cell cycle by miR-20a-5p inhibitor was reversed by pcDNA3.1-CACF, and the deceleration of the progression of cell cycle by miR-20a-5p mimic was reversed by CACF silencer in vascular cells (Figure 5V). Flow cytometry also revealed that the inhibition of pcDNA3.1-CACF on apoptosis was reversed by miR-520b-3p mimic, and the promotion of CACF silencer on apoptosis was reversed by miR-520b-3p inhibitor (Figure 5W). The inhibition of miR-20a-5p inhibitor on apoptosis was reversed by pcDNA3.1-CACF but the promotion of miR-20a-5p mimic on apoptosis was reversed by CACF silencer (Figure 5).

### MiR-520b-3p and miR-20a-5p inhibited autophagy in vascular cells

**Figure 6:**
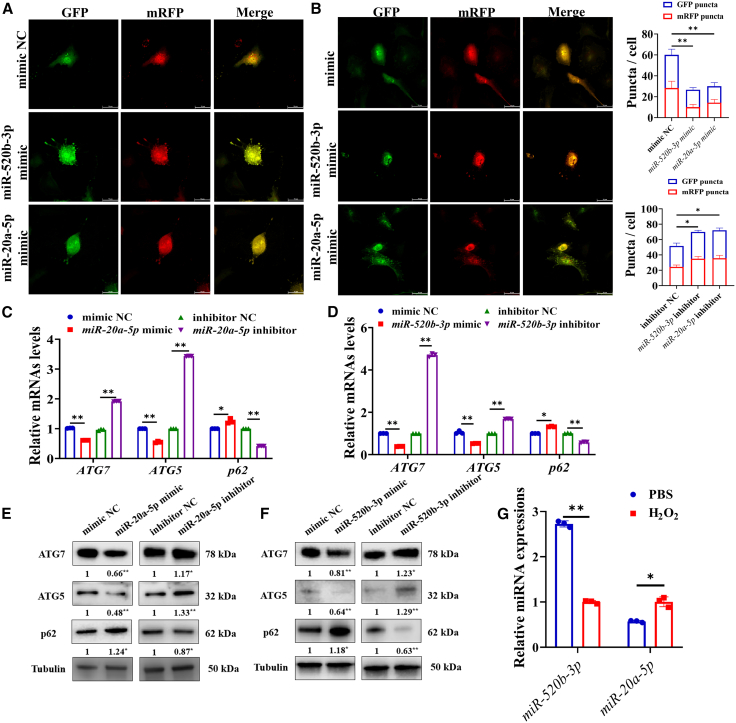
MiR-520b-3p and miR-20a-5p inhibited autophagy in vascular cells (A) Effects of miR-520b-3p mimic and miR-20a-5p mimic on the autophagy in vascular cells. (B) Effects of miR-520b-3p inhibitor and miR-20a-5p inhibitor on autophagy in vascular cells (scale bars, 100 μm). Effects of miR-520b-3p (C) and miR-20a-5p (D) on the expression levels of autophagy marker genes in vascular cells. Effects of miR-520b-3p (E) and miR-20a-5p (F) on the expression levels of autophagy marker proteins in vascular cells. (G) Expressions of miR-520b-3p and miR-20a-5p in the hypoxic environment of MI simulated by H_2_O_2_. Data are shown as mean ± SEM; ∗*p* < 0.05, ∗∗*p* < 0.01; unpaired two-tailed Student’s *t* test. Scale bars, 100 μm in (A and B); *n* = 3 biological replicates per group.

We found both miR-520b-3p mimic and miR-20a-5p mimic significantly reduced the formation of autophagic flux (Figure 6A), while miR-520b-3p inhibitor and miR-20a-5p inhibitor significantly increased the formation of autophagic flux (Figure 6B). RT-qPCR showed that both miR-520b-3p and miR-20a-5p led to a significant downregulation in the expression levels of autophagy marker genes, including *ATG7* (*p* < 0.01), *ATG5* (*p* < 0.01), and upregulated the mRNA level of *p62* (*p* < 0.05) (Figures 6C and 6D). Western blot showed that both miR-520b-3p and miR-20a-5p significantly decreased the levels of autophagy marker proteins, including ATG7 (*p* < 0.01) and ATG5 (*p* < 0.01) (Figures 6E and 6F). Conversely, there was an observable increase in the level of p62 (*p* < 0.05), which degraded during autophagy (Figures 6E and 6F). Furthermore, we simulated the hypoxic environment of cardiac vascular cells during MI by using H_2_O_2_, and observed the expression of miR-520b-3p was significantly downregulated, whereas the expression of miR-20a-5p was notably upregulated (Figure 6G).

### CACF regulate autophagy to promote cardiac recovery after MI

**Figure 7:**
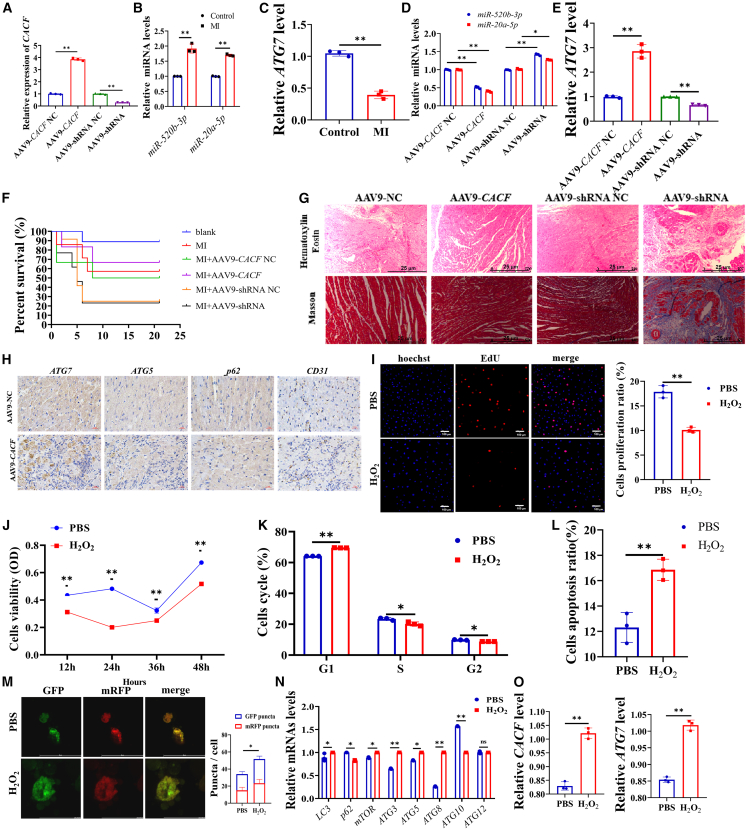
CACF regulated autophagy to promote cardiac recovery after MI (A) The level of CACF in rat hearts with MI after the injection of AAV9-CACF/NC and AAV9-shRNA/NC. The levels of miR-520b-3p and miR-20a-5p in the hearts of MI rats (B), and the level of ATG7 in the hearts of MI rats (C). The levels of miR-520b-3p and miR-20a-5p in the hearts of MI rats (D), and the level of *ATG7* in the hearts of MI rats (E) after the injection of AAV-9-CACF/NC or AAV9-shRNA/NC. (F) The survival rate of rats with MI in each treatment group. (G) Changes of histological and pathological in the hearts of rats with MI after the injection of AAV-9-CACF/NC or AAV9-shRNA/NC. (H) The protein levels of ATG7, ATG5, p62, and CD31 in the hearts of rats with MI after the injection of AAV-9-CACF/NC or AAV9-shRNA/NC. Effect of hypoxic conditions on proliferation (I), viability (J), cell cycle (K), and apoptosis (L) of vascular cells. (M) Effect of hypoxic conditions on autophagy of vascular cells. (N) The expressions of autophagy marker genes in vascular cells during hypoxic conditions. (O) The levels of CACF and *ATG7* in vascular cells during hypoxic conditions. Data are shown as mean ± SEM; ∗*p* < 0.05, ∗∗*p* < 0.01; unpaired two-tailed Student’s *t* test. Scale bars: 25 μm in (G and H); 100 μm in (I); and 50 μm in (M); *n* = 3 biological replicates per group.

To explore the therapeutic potential of CACF in enhancing cardiac recovery after MI, we employed an adenovirus-associated virus (AAV)-9 vector to deliver CACF and specific short hairpin RNA (shRNA) into the rat MI model. RT-qPCR showed a marked upregulation of CACF in the myocardium of rats treated with AAV9-CACF, whereas a notable reduction was observed in the AAV9-shRNA group (Figure 7A). We found the expressions of miR-520b-3p and miR-20a-5p were significantly elevated in the hearts of MI rats (Figure 7B), and the expression of *ATG7* was decreased in the hearts of MI rats (Figure 7C). However, the expressions of miR-520b-3p and miR-20a-5p were decreased by AAV9-CACF (Figure 7D), and the expression of *ATG7* was increased by AAV9-CACF (Figure 7E).

We also found that AAV9-CACF significantly enhanced the survival rates (Figure 7F). Furthermore, AAV9-CACF significantly reduced the disorganized myocardial collagen fibers, while AAV9-shRNA was opposite (Figure 7G), compared to control groups. Moreover, AAV9-CACF significantly increased the protein levels of ATG7 and ATG5, but decreased the protein level of p62, compared to the control group (Figure 7H). Furthermore, the protein level of CD31, which played a crucial role in angiogenesis, was significantly elevated in the AAV9-CACF group (Figure 7H).

Vascular cells were exposed to H_2_O_2_ to simulate the hypoxic conditions in MI. We found the proliferation, viability and cell cycle of vascular cells were significantly inhibited (Figures 7I–7K), while the apoptosis was significantly promoted (Figure 7L). The autophagy of vascular cells was also significantly enhanced (Figure 7M), and the expression levels of autophagy marker genes were significantly elevated, including *LC3* (*p* < 0.05), *ATG3* (*p* < 0.05), *ATG5* (*p* < 0.01), *ATG8* (*p* < 0.05), and *mTOR* (*p* < 0.01), with a concurrent decrease in *p62* (*p* < 0.05) (Figure 7N). This treatment also led to a significant upregulation of CACF and *ATG7* in vascular cells (Figure 7O).

## Discussion

LncRNAs have been reported to play crucial roles in cardiovascular physiology and pathology.^32^ We found five lncRNAs significantly downregulated in MI tissues, compared to normal cardiac tissues in a pig model of MI (Figure 1C), and these five lncRNAs were also expressed in the human genome (Figure 1D). Numerous studies have shown that vascular regeneration could be an effective approach for treating MI, and the vascular endothelial cells are widely recognized as a standard cell model for studying vascular regeneration.^33^^,^^34^ To focus on the function of lncRNAs in vascular endothelial cells, the overexpression vectors and corresponding silencers were engineered to investigate their effects in human umbilical vein endothelial cells (HUVECs) for each lncRNA. Our findings showed that lncRNA3 notably enhanced angiogenesis (Figure 1F), while it significantly promoted the proliferation and inhibited the apoptosis of vascular cells (Figures S1A and S1B). Due to the low accuracy of *trans*-regulation prediction for lncRNAs, we predicted the *cis*-regulatory target genes of lncRNAs based on their positional relationships. Differential mRNAs and differential lncRNAs involved in *cis*-regulation were then subjected to predictive analysis,^35^ and we found that lncRNA3 might altered the mRNA and protein levels of *ATG7* (Figure 1G). *ATG7* has been demonstrated to regulate cardiac recovery after MI through autophagy, and lncRNA3 changed both the mRNA and protein levels of *ATG7*, so we named lncRNA3 as the CACF. Although pigs exhibit high sequences and chromosomal structural homologies with humans, and share similarities in physiology, organ development and disease progression, and making them a primary mammalian model for human research,^36^ the pig MI model might not fully show the complexity of human heart disease. It was important to note that, despite our stringent control of potential variables during the construction of the MI pig model, the differences of genetic background and variations in the expressions of genes among individuals and certain subtle environmental factors might still inevitably influenced the experimental results. We aimed to compare the relative impact of five lncRNAs on vascular cells, and the inclusion of a negative control group could potentially strengthen the persuasive power of our data.

The proliferative capacity of cardiac cells in adult mammals is very limited, leading to a disappointing prognosis for MI,^37^ and timely regeneration of myocardial vessels is critical for cardiac recovery following MI. The proliferation ability of vascular cells directly impacts angiogenesis.^38^ Similarly, our results demonstrated that CACF significantly promoted the proliferation (Figures 2A–2F), while reduced the apoptosis rate of vascular cells (Figures 2H–2J). Due to the observation of similar progression in rat cardiac disorders and heart failure as seen in patients with heart failure, the rat MI model is often used to simulate human cardiovascular diseases and evaluate the contributions of treatment strategies to heart failure therapy.^39^ Left anterior descending artery ligation is a commonly used method to establish a rat MI model. We established a rat MI model using left anterior descending artery ligation (Figures 3A and 3B). This technique allows for easier localization of the left anterior descending artery, accurately induces left ventricular ischemia in the rat heart, and enhances the reproducibility of the infarct area.^40^ We found that the level of CACF in the hearts of MI rat showed a similar downregulation as observed in the hearts of MI pig (Figure 3C), and it was highly conserved across human, rat, and pig hearts (Figure 3D).

Several studies have reported the regulation role of lncRNAs in inductions on autophagy in MI. For example, lncRNA *H19* reduces infarct size and improves related cardiac function by promoting autophagy,^17^ whereas lncRNA *Galont* upregulates the expression of *ATG5*, leading to excessive autophagy and triggering cell apoptosis, which results in poorer cardiac recovery.^41^ But lncRNA CAIF significantly reduces autophagy, decreases autophagy-induced myocardial cell death, and improves cardiac function.^42^ We found that CACF significantly enhanced autophagy in vascular cells, and enhanced the levels of *ATG7* (Figures 3F–3H). *ATG7* is a well-established autophagy marker that has been linked to various pathologies, including cancer, neurological, and cardiovascular diseases.^43^^,^^44^ Recent studies have revealed that *ATG7* plays a critical role in promoting autophagy and inhibiting apoptosis, contributing to the anti-aging effects in vascular cells and protecting cardiomyocytes from ischemia-reperfusion injury.^45^^,^^46^ Similarly, we discovered that *ATG7* facilitated the proliferation and the progression of cell cycle of vascular cells (Figures 4B–4E). Generally, the increased level of autophagy is often associated with higher rates of cell apoptosis.^47^ Our results showed that *ATG7*, as an autophagy marker, inhibits apoptosis in vascular cells (Figure 4F). This might be due to the non-autophagic pathways regulated by *ATG7*.^48^ Moreover, we found that the presence of binding sites for miR-520b-3p and miR-20a-5p on CACF and *ATG7*, and CACF regulated the expression of *ATG7* by competitively binding to miR-520b-3p and miR-20a-5p (Figures 4I and 4L). Compared with other autophagy pathways, such as those mediated by mechanistic target of rapamycin (mTOR) signaling pathway or Adenosine 5′-monophosphate-activated protein kinase (AMPK) signaling pathway, CACF might function in a more specific and localized manner, directing autophagic activity toward key cellular components essential for cardiac regeneration, thereby exerting a positive effect by promoting vascular cell proliferation and inhibiting apoptosis.

MicroRNAs play key roles in multiple cardiovascular diseases,^49^^,^^50^ including MI,^51^ heart failure^52^ and atherosclerosis,^53^ and have been shown to facilitate the recovery of heart function after ischemic injury.^54^ In this study, we also focused miR-520b-3p and miR-20a-5p on the functions of vascular cells to explore their broader applications in MI. MiR-520b-3p is implicated in managing the cellular stress response in oncology settings,^55^ and miR-20a-5p has been identified as targeting ferroptosis to mitigate renal ischemic damage dependent on long-chain acyl-CoA synthetase 4 (ACSL4).^56^ However, their biological functions in the context of MI have yet to be investigated. Our findings showed that miR-520b-3p inhibited proliferation (Figure 5A) and promoted apoptosis (Figure 5I) of vascular cells, while miR-20a-5p promoted proliferation (Figure 5B) and inhibited apoptosis of vascular cells (Figure 5J). Interestingly, both miR-520b-3p and miR-20a-5p inhibited the autophagy in vascular cells (Figures 6A and 6B). We thought that the discrepancy in the effects of miR-520b-3p and miR-20a-5p on apoptosis and autophagy in vascular cells might be attributed to the difference in the degree of autophagy induction or the dual targeting of CACF. The dual targeting of CACF might help balance the contrasting effects of these two miRNAs, thereby optimizing vascular repair. By modulating the interaction between CACF and miR-520b-3p/miR-20a-5p, it might precisely regulate the vascular repair process, reduced cell death and promoting the reconstruction of healthy blood vessels.

Hydrogen peroxide is commonly used to construct hypoxia models in cells to simulate the hypoxic conditions experienced by vascular cells during MI,^57^ and we found that under hypoxic conditions, the proliferative of vascular cells was significantly reduced (Figure 7I), accompanying by higher levels of apoptosis (Figure 7L) and autophagy (Figure 7M), while the expressions of both *CACF* and *ATG7* were significantly increased (Figure 7O), which concurrently promoted the expressions of autophagy marker genes in vascular cells (Figure 7N). The bidirectional effect of miR-520b-3p and miR-20a-5p on HUVECs phenotype suggests that the function of miRNAs within cells is not singular. We simulated the hypoxic environment that cells experience after MI by treating HUVECs with hydrogen peroxide. Unlike the MI rat model, we observed a significant upregulation of miR-520a-3p following hydrogen peroxide treatment. The possible explanation for this is that, after hydrogen peroxide treatment, other hypoxic or oxidative stress pathways involving miR-520b-3p are activated, leading to important regulatory effects on its expression. This observation was both intriguing and warrants further attention. We observed that both the MI + AAV9-shRNA and MI + AAV9-shRNA-NC groups exhibited approximately 80% 1 week mortality, which might be attributed to the immunogenicity induced by AAV and the adverse prognosis associated with MI. Our assessments further demonstrated that CACF significantly enhanced cardiac recovery and survival following MI in rat models (Figures 7F and 7G). We observed a decrease in the expressions of miR-520b-3p and miR-20a-5p, along with an increase in the expression of *ATG7* induced by CACF (Figures 7B–7E). Moreover, the protein levels of ATG7, ATG5, and CD31 were significantly elevated by CACF in the rat hearts following MI (Figure 7H). These findings suggested that CACF might regulate autophagy to enhance cardiac recovery by competitively binding to miR-520b-3p and miR-20a-5p.

Our study found that CACF upregulated *ATG7* and enhanced autophagy by competitively binding to miR-20a-5p and miR-520b-3p, which accompanied by improved cardiac function following MI. However, further investigation is needed to the safety of CACF-mediated mechanisms in human MI patients through studies involving human patients or clinical sample data, which was one of the limitations of our study. Our findings provided new insights and a research foundation for the role of *ATG7* in influencing cardiac recovery after MI through autophagy, and had the potential to become a new biomarker of the diagnosis for MI. However, the high mortality rate observed in rats following AAV delivery suggested limitations of traditional delivery systems. As a potential therapeutic target, the application of CACF was hindered by the instability, negative charge, and hydrophilic nature of lncRNAs, which restricted its *in vivo* use. The use of alternative non-viral delivery systems, such as lipid nanoparticles or inorganic nanoparticles, may have been crucial for the future translation of *CACF*-based therapies, and further investigation is needed to the safety of *CACF*-mediated mechanisms in human MI patients through studies involving human patients or clinical sample data. Overall, our study offered a potentially viable strategy and providing new perspectives and references for research on MI treatment.

### Limitations of the study

The alteration of cardiac function was inevitably influenced by multi-gene regulation. Because *ATG7* directly participated in autophagosome formation, *CACF*-mediated upregulation of *ATG7* might specifically target ischemia-damaged cardiomyocytes or injured vascular cells, facilitating the clearance of particular damaged organelles or proteins, rather than broadly regulating autophagic activity through alterations in cellular energy metabolism or stress responses. We observed that *ATG5* was significantly upregulated by CACF, and we speculated that this was due to *ATG7* activating *ATG12*, which facilitated the binding of *ATG12* to *ATG5* to form the *ATG12*-*ATG5* complex. Other *ATGs* might also follow similar pathways. The key regulatory mechanisms of other autophagy-related molecules in MI remained to be further explored.

## Resource availability

### Lead contact

Further information and requests for resources and reagents should be directed to and will be fulfilled by the lead contact, Xiaolong Yuan (yxl@scau.edu.cn).

### Materials availability

This study did not generate new unique reagents.

### Data and code availability

• All data reported in this article will be shared by the lead contact upon request.
• This article does not report original code.
• Any additional information required to reanalyze the data reported in this article is available from the lead contact upon request.

## Acknowledgments

This research was supported by the 10.13039/501100001809National Natural Science Foundation of China (32070542), the 10.13039/501100021171Guangdong Basic and Applied Basic Research Foundation (2021A1515010873 and 2022A1515011455), and the Breed Industry Innovation Park of Guangdong Xiaoerhua Pig (2022-4408X1-43010402-0019). The authors would like to express their gratitude for the financial support that made this study possible.

## Author contributions

J.Y., data curation, formal analysis, investigation, methodology, validation, visualization, writing – original draft, and writing – review and editing; G.B., data curation, formal analysis, and investigation; W.L., Q.Z., N.L., X.Z., Y.H., H.Q., J. Zhou, C.L., Y.G., E.H., L.Z., L.Y., J. Zeng, and H.Z. formal analysis, investigation, and methodology; X.Y., conceptualization, funding acquisition, methodology, project administration, resources, software, supervision, and writing – review and editing; X.W., conceptualization, funding acquisition, methodology, project administration, resources, software, supervision, and writing – review and editing.

## Declaration of interests

The authors declare that they have no competing interests.

## Declaration of generative AI and AI-assisted technologies in the writing process

During the preparation of this work, we used ‘‘DeepSeek’’ AI to improve the logical coherence of the language. After using this tool, we reviewed and edited the content as needed and take full responsibility for the content of publication.

## STAR★Methods

### Key resources table

**Table:** 

REAGENT or RESOURCE	SOURCE	IDENTIFIER
**Antibodies**		

MCL1	Abcam	Ab32087; RRID:AB_776245
PCNA	Abcam	Ab92552; RRID:AB_10561973
CD31	Abcam	Ab182981; RRID:AB_2920881
CASP8	Abcam	Ab108333; RRID:AB_10866391
p53	Abcam	Ab32049; Ab32049
ATG7	Abcam	Ab133528; RRID:AB_2532126
ATG5	Abcam	Ab108327; RRID:AB_2650499
p62	Abcam	Ab109012; RRID:AB_2810880
LC3	Abcam	Ab192890; RRID:AB_2827794
CASP3	Abcam	Ab184787; RRID:AB_2827742
CASP7	Abcam	Ab32522; RRID:AB_725952
CASP9	Abcam	Ab202069
CCND1	Abcam	Ab134175
CCNE1	Abcam	Ab238081
BECN1	Abcam	Ab114071
GAPDH	Abcam	Ab181062
Tubulin	Abcam	Ab179513
goat anti-mouse	Abcam	Ab6789
goat anti-rabbit IgG	Abcam	Ab205718

**Bacterial and virus strains**		

Adeno-associated virus vectors	Dongze Biotechnology Co., Ltd	N/A

**Biological samples**		

Male Juema minipigs	Guangdong Provincial Biotechnology Research Institute	N/A
Male SD rats	Guangdong Medical Laboratory Animal Center	N/A

**Chemicals, peptides, and recombinant proteins**		

TRIzol	Thermo	15596018CN
Endothelial cell culture medium	ScienCell	#0025
Fetal bovine serum	ScienCell	#0500
HEPES	Thermo	15630106
RevertAid First Strand cDNA Synthesis Kit	Thermo	K1621
SYBR Green RT‒qPCR Mix (2×)	Thermo	K0222
Cell-LightTM EdU Apollo Kit	Ribo Bio	C10310-1
GFP-mRFP-LC3	Hanheng Bio	HB-AP210 0001
Cell Counting Kit-8	Sewen Bio	Sc119
BCA Protein Assay Kit	Beyotime	P0012

**Deposited data**		

RNA-seq data	This paper	PRJNA1424201

**Experimental models: Cell lines**		

Human umbilical vein endothelial cells	ScienCell	#8000

**Experimental models: Organisms/strains**		

Male Juema minipigs	Guangdong Provincial Biotechnology Research Institute	N/A
Male SD rats	Guangdong Medical Laboratory Animal Center	N/A

**Oligonucleotides**		

Primers for this study	Tsingke Bio	N/A
Small interfering RNAs	Ribo Bio	N/A
Control pcDNA3.1plasmid, ATG7 and CACF Overexpression plasmid	Ribo Bio	N/A
The miR-520b-3p/miR-20a-5p mimic, inhibitor and NC.	Ribo Bio	N/A

**Software and algorithms**		

ImageJ	National institutes of health	http://ImageJ.nih.gov/ij
GraphPad Prism	GraphPad Software, San Diego, CA, USA	Version 8.0.2

### Experimental model and study participant details

Six Male Juema minipigs (10 months of age, weight 20–25 kg) were raised in Guangdong Provincial Biotechnology Research Institute (Guangdong Provincial Laboratory Animals Monitoring Center).and sixty Male SD rats (7–8 weeks of age, weight 220 ± 20 g) were purchased from the Guangdong Medical Laboratory Animal Center were used for the study. All the animals were fed under controlled temperature and light (about 22 ± 2°C, 12 h light/dark cycle) conditions and were allowed free access to food and water. The left anterior descending (LAD) artery of the heart of the Juema minipigs was applied promptly to occlude by a coronary artery occlusion device. The LAD of the SD rat was blocked with a surgical thread. Animals were euthanized immediately after echocardiographic examinations, and heart tissues were collected. All experimental protocols were approved by the Ethics Committee of Guangdong Provincial Biotechnology Research Institute (Guangdong Provincial Laboratory Animals Monitoring Center) (IACUC2021151). All methods are reported in accordance with the ARRIVE guidelines to ensure transparency and reproducibility in animal research.

### Method details

#### Establishment of the MI animal model

Full-body anesthesia was induced in miniature pigs placed in the supine position on an operating table and mechanically ventilated. Skin incisions were made at the fourth left rib, and after gradual dissection through the skin and subcutaneous tissue, the pericardium was exposed and locally anesthetized with 5% lidocaine. The left atrial appendage was retracted, and the origin of the second diagonal branch along the left anterior descending (LAD) artery was identified. Following incision of the external membrane, approximately 1.5 cm of the LAD vessel was exposed. Two sutures were used to gently retract the vessel, and a coronary artery occlusion device was applied promptly to occlude the vessel. The anterior wall of the left ventricle gradually became ischemic, evidenced by a dark red color and reduced motion. Subsequently, the LAD artery was ligated, ensuring no active bleeding before meticulous layer-by-layer suturing [19]. The pigs in control group underwent thoracotomy only without ligation. The Bene View T5 electrocardiogram monitoring system and the EDAN H100 monitor (Edan Instruments, Inc., China) were used to monitor the basic vital signs of animals during the operation. Intramuscular injection of penicillin is used to prevent postoperative infection. Male Sprague-Dawley (SD) rats weighing 220 g were procured from the Guangdong Medical Laboratory Animal Center. 60 SD rats were randomly divided into two groups: blank group (*n* = 10) and MI group (*n* = 50). To anesthetize the rats, each rat received an intramuscular injection of 0.2% sumiaxin-ketamine per kilogram body weight. Following anesthesia, a thoracotomy was performed to expose the left ventricle, and the LAD artery was ligated between the pulmonary artery outflow tract and the left atrium. The blank group underwent thoracotomy without ligation. 50 rats from MI group were randomly divided into five groups: MI group (*n* = 10), MI + AAV9-CACF NC group (*n* = 10), MI + AAV9-CACF group (*n* = 10), MI + AAV9-shRNA group (*n* = 10), and MI + AAV9-shRNA NC group (*n* = 10). Adenoviruses were injected to the site of ischaemia in the heart of rats in each group via cardiac *in situ* injection postoperatively, with the following doses: 2 × 10^11^ MOI adeno-associated virus of CACF, 2 × 10^11^ MOI adeno-associated virus of CACF NC, 2 × 10^10^ MOI adeno-associated virus of CACF-shRNA, and 2 × 10^10^ MOI adeno-associated virus of shRNA NC, administered weekly. The adeno-associated virus vectors were synthesized by Dongze Biotechnology Co., Ltd. (Guangzhou, China). Weekly administration continued for three weeks, and echocardiographic examinations were performed using a Vevo 2100 system (VisualSonics Inc, Toronto, Canada) equipped with an 80 MHz probe [20].

#### RNA sequencing

RNA was extracted from infarcted tissue of pigs with myocardial infarction and the corresponding regions of normal pigs using TRIzol (Thermo, Massachusetts, US). Samples were stored at −80°C until use. RNA quality was assessed using a Nanodrop 2000 spectrophotometer (Thermo, Massachusetts, US), with an A260/A280 ratio between 1.8 and 2.0 considered acceptable. Subsequently, RNA sequencing was performed using an Illumina HiSeq 2500. Adapter sequences were removed using Cutadapt 3.5, and quality trimming was performed with Trimmomatic 0.39. The Sus scrofa 11.1 genome was used as the reference for alignment, with HISAT2 2.2.1 for alignment and Stringtie 2.1.4 for transcript assembly. Coding potential was predicted using CPC2 2.0, CNCI 2.0, and FEELnc 1.0. Transcripts predicted as non-coding and longer than 200 nt were classified as lncRNAs. Differentially expressed genes were conducted using the DESeq2 1.36.0 package. Data preprocessing included normalization and filtering, with significance criteria set to |log2FC| > 1 and FDR <0.05.

#### Cell culture and treatment

Human umbilical vein endothelial cells (HUVECs) obtained from ScienCell (California, USA) were cultured in endothelial cell culture medium (ECM, California, ScienCell) supplemented with 5% fetal bovine serum (FBS, California, ScienCell), 1% penicillin-streptomycin, and 1% endothelial cell growth factors (ECGS, California, ScienCell). Cells were maintained at 37°C in a 5% CO2 humidified atmosphere, with medium replenished every 48 h. Transfections were performed using Lipofectamine 3000 (Thermo Scientific, USA). The pcDNA3.1-CACF group served as the treatment group, with the pcDNA3.1 group as its control group. The CACF Silencer group was the treatment group, and the Silencer NC group as its control group. The miR-520b-3p mimic group was the treatment group, with the mimic NC group as its control group. The miR-520b-3p inhibitor group was the treatment group, and the inhibitor NC group as its control group. The miR-20a-5p mimic group was the treatment group, with the mimic NC group as its control group. The miR-20a-5p inhibitor group was the treatment group, and the inhibitor NC group as its control group. The pcDNA3.1-ATG7 group served was the treatment group, with the pcDNA3.1 group as its control group. The siRNA-ATG7 group was the treatment group, and the siRNA-NC group as its control group.

#### Nuclear-cytoplasmic separation assay

Vascular cells were cultured to 80% confluency, then washed with PBS buffer. Next, lysis buffer which containing 20 mM HEPES (Thermo Scientific, USA), 1.5 mM MgCl_2_, and 0.5% Triton X-100 was added, and the mixture was incubated on ice for 10 min to disrupt the cell membrane. Subsequently, the cells were centrifuged at 3000 rpm for 5 min to separate the cytoplasm and nuclei. The supernatant was collected as the cytoplasmic sample, and the pellet was obtained as the nuclear fraction.

#### *In vitro* tube formation assay

The matrix gel was mixed thoroughly on ice, and 50 μL of matrix gel was added to each well to cover the bottom. The plate was placed in a 37°C cell culture incubator to melt the matrix gel. The cell suspension was prepared, added to the 96-well plate, and then incubated at 37°C and 5% CO2 for 6 h in a cell culture incubator, followed by observation under a phase-contrast microscope (Nikon, Tokyo, Japan).

#### Real-time quantitative PCR

Total RNA was extracted from samples using TRIzol reagent (TaKaRa, Kyoto, Japan). Subsequently, cDNA was synthesized using the RevertAid First Strand cDNA Synthesis Kit (Thermo Fisher Scientific, Massachusetts, USA). Quantitative PCR was conducted using SYBR Green RT‒qPCR Mix (2×) (Thermo Fisher Scientific, MA, USA) on a CFX96 Touch PCR instrument (Bio‒Rad, Berkeley, USA). Glyceraldehyde phosphate dehydrogenase (GAPDH) or U6 small nuclear RNA served as endogenous controls, and the 2−ΔΔct method was employed to analyze expression levels. Primer sequences are detailed in Table S1.

#### EdU assay

EdU assays were performed according to the manufacturer’s instructions using the Cell-LightTM EdU Apollo Kit (Ribo Bio, Guangzhou, China). HUVECs transfected with plasmids were seeded in 48-well plates and treated with Triton X-100 for 10–15 min after 24 h. Subsequently, cells were incubated with EdU staining buffer for 30–45 min followed by Hoechst staining for 10–15 min. Proliferation rates were determined by calculating the ratio of EdU-labeled proliferating cells to Hoechst-labeled cells in three random fields per well under an inverted fluorescence microscope. The images were observed using the ECLIPSE Ti2 Series (Nikon, Tokyo, Japan) inverted fluorescence microscope with a 20× objective. The excitation wavelength was 643 nm for red (EdU), and 360 nm for blue (DAPI). Images were captured using NIS-Elements BR (Nikon, Tokyo, Japan).

#### GFP-mRFP-LC3 analysis

HUVECs were seeded in 24-well plates and cultured until reaching 50% confluence. GFP-mRFP-LC3 (Hanheng Bio, Beijing, China) at an MOI of 15 was added to each well, and after 12 h, the culture medium was replaced. Transfection proceeded for an additional 12 h before fixing cells with 4% formaldehyde for 10 min. After PBS washing, cells were observed using a confocal microscope (Nikon, Tokyo, Japan) at 100× magnification.

#### CCK8 assay

Cell viability was assessed using the Cell Counting Kit-8 (CCK-8) (Qiwen Bio, Shanghai, China). HUVECs were seeded in 96-well plates and transfected when cell density reached 70%. At 12, 24, 36 and 48 h post-transfection, 10 μL of CCK-8 reagent (5 mg/mL) was added to each well. Following a 2-h incubation, absorbance at 570 nm was measured using a multifunctional enzyme marker (Promega, Madison, US) to determine cell viability.

#### Flow cytometry

Following the instructions provided in the Annexin V-FITC Apoptosis Detection Kit from Bio Vision, USA, HUVECs transfected with plasmids in 6-well plates for 24 h were resuspended in 1× Annexin V buffer. Subsequently, the cells were treated with Annexin V-FITC and propidium iodide (PI) in the dark. The apoptosis of HUVECs was analyzed using flow cytometry (Guava, Luminex, US), with the results indicating the combined sum of cells in the early and late apoptosis stages.

#### Western blotting

The concentration of total protein extracted from the samples was determined using the BCA Protein Assay Kit (Beyotime, Nanjing, China). Equal amounts of protein from each sample were electrophoresed on SDS‒PAGE gels and transferred to polyvinylidene difluoride (PVDF) membranes. The PVDF membranes were treated with 5% skim milk at 37°C for 1 h and then incubated with antibodies against myeloid cell leukemia sequence 1 (MCL1, Abcam, 1:1000), proliferating cell nuclear antigens (PCNA, Abcam, 1:1000), platelet endothelial cell adhesion molecule-1 (CD31, Abcam, 1:1000), cysteinyl aspartate specific proteinase 8 (CASP8, Abcam, 1:5000), tumor Protein 53 (p53, Abcam, 1:2000), autophagy related protein 7 (ATG7, Abcam, 1:1000), autophagy related protein 5 (ATG5, Abcam, 1:1000), sequestosome 1 (p62, Abcam, 1:5000), microtubule-associated protein 1 light chain 3 (LC3, Abcam, 1:2000), cysteinyl aspartate specific proteinase 3 (CASP3, Abcam, 1:2000), cysteinyl aspartate specific proteinase 7 (CASP7, Abcam, 1:3000), cysteinyl aspartate specific proteinase 9 (CASP9, Abcam, 1:10,000), cyclin D1 (CCND1, Abcam, 1:1000), cyclin E1 (CCNE1, Abcam, 1:1000), recombinant beclin 1 (BECN1, Abcam, 1:1000), GAPDH (Abcam, 1:10000), and Tubulin (Abcam, 1:10000) at 4°C overnight. The membranes were washed and then incubated with either goat anti-mouse (ab6789, Abcam, 1:5000) or goat anti-rabbit IgG H&L (ab205718, Abcam, 1:10000) at 37°C for 2 h. A Tanon 5200 Muti instrument (Shanghai, China) was used to observe the blots, and ImageJ software was used for analysis. Some panels were performed using the same membrane for multiple detections, or through stripping and reprobing (Thermo Fisher Scientific, Massachusetts, USA).

#### RNA pull-down

HUVECs were lysed using standard lysis buffers as per the protocol of the PierceTM Magnetic RNA‒Protein Pull-Down Kit (Thermo Fisher Scientific). The 3′ end of the target long noncoding RNA (lncRNA) was biotin-labeled and bound to streptavidin magnetic beads. The RNA-bound beads were treated with 1× RNA capture buffer and 100 μL of 1× protein‒RNA-binding solution, followed by incubation with 100 μL of Master Mix for 30–60 min. The RNA-binding complexes were subsequently eluted from the beads.

#### Dual-luciferase assay

The potential binding relationships between CACF, miR-520b-3p, miR-20a-5p, and the ATG7 3′ UTR were predicted using the website http://www.targetscan.org/. The predicted binding sequences on the 3′ UTRs of CACF and ATG7 were synthetically generated through PCR and then transferred into a luciferase reporter vector from Promega, USA. Vein endothelial cells were seeded in 96-well plates for the experiment. A total of 100 ng of the CACF and ATG7 3′ UTR luciferase reporter plasmids, along with either 50 nM of miR-520b-3p and miR-20a-5p mimic oligonucleotide or a nontargeting miRNA mimic control (negative control), were co-transfected into the cells for 24 h. The luciferase activity in each well was subsequently measured using the Dual Glo Luciferase Assay System from Promega, USA, following the manufacturer’s instructions.

#### Immunohistochemical staining

Heart sections from rats were dried, dewaxed in xylene, rehydrated with alcohol, and treated with citrate buffer (pH 6.0) followed by autoclaving. After washing with PBS, sections were blocked with goat serum (Boster) and then incubated overnight at 4°C with antibodies against ATG7, ATG5, p62, and LC3 (Abcam, Cambridge, UK) diluted at 1:200. After washing with PBS, sections were incubated with the Polink-1 HRP DAB Detection System (ZSGB-BIO, Beijing, China) for 20 min at room temperature. Sections were examined using an inverted fluorescence microscope (Leica, Wetzlar, Germany).

#### Hematoxylin and eosin (H&E) staining

To prepare the hearts for histological analysis, they were fixed in 4% paraformaldehyde. After fixation, the hearts were embedded in paraffin and cut into 4 μm sections. The dewaxed sections were then incubated with hematoxylin for 5 min to stain the nuclei and eosin dye solution for 3 min to provide contrast to the tissues. Following staining, the sections were dehydrated and sealed to preserve the samples. Finally, the sections were photographed under a fluorescence microscope to visualize the cellular structures and any fluorescence signals present in the samples.

#### Masson staining

The experimental procedure involved the fixation of the hearts in 4% paraformaldehyde, followed by embedding in paraffin and cutting into 4 μm sections for histological analysis. The dewaxed sections were initially incubated with hematoxylin for 5 min to stain the nuclei, followed by incubation with Ponceau S for 5 min. Subsequently, the sections were treated with a phosphomolybdate aqueous solution for 5 min and then dyed with aniline blue for 5 min to achieve the desired staining. After the staining process, the sections were dehydrated and sealed following treatment with 1% acetic acid for 1 min. Finally, the sections were examined under an optical microscope from Leica (Wetzlar, Germany) to observe and analyze the cellular structures and staining patterns.

### Quantification and statistical analysis

The image processing and segmentation were performed using ImageJ software. The statistical analyses were conducted using Prism software from GraphPad (San Diego, US). Data were expressed as the mean ± SEM of at least three independent experiments per cell treatment group and at least five independent experiments per animal group. We evaluated the data with unpaired Student’s *t* test or multiple comparisons, one-way ANOVA. ∗*p* < 0.05, ∗∗*p* < 0.01; A *p*-value of <0.05 was considered significant.
