# Supplementary material for: LncRNA CACF promotes autophagy and cardiac recovery after myocardial infarction by targeting ATG7
Source: iScience. 2026 Apr 28;29(6):115889. doi: 10.1016/j.isci.2026.115889 (PMC13206723; doi:10.1016/j.isci.2026.115889)
Supplement: Document S1. Figure S1 and Tables S1 and S2 [file mmc1.pdf]

**iScience, Volume *29***

## Supplemental information

# LncRNA *CACF* promotes autophagy and cardiac recovery after myocardial infarction by targeting *ATG7*

**Jinghao Yang, Guofeng Bai, Weili Liao, Qingyang Zhao, Nian Li, Xiaofeng Zhou, Yingting He, Jingyu Zhou, Hongyan Quan, Chennan Lin, Yixuan Guo, Liuhong Zhang, Enyuan Huang, Linfang Yang, Jianghua Zeng, Hao Zhang, Xiaolong Yuan, and Xilong Wang**

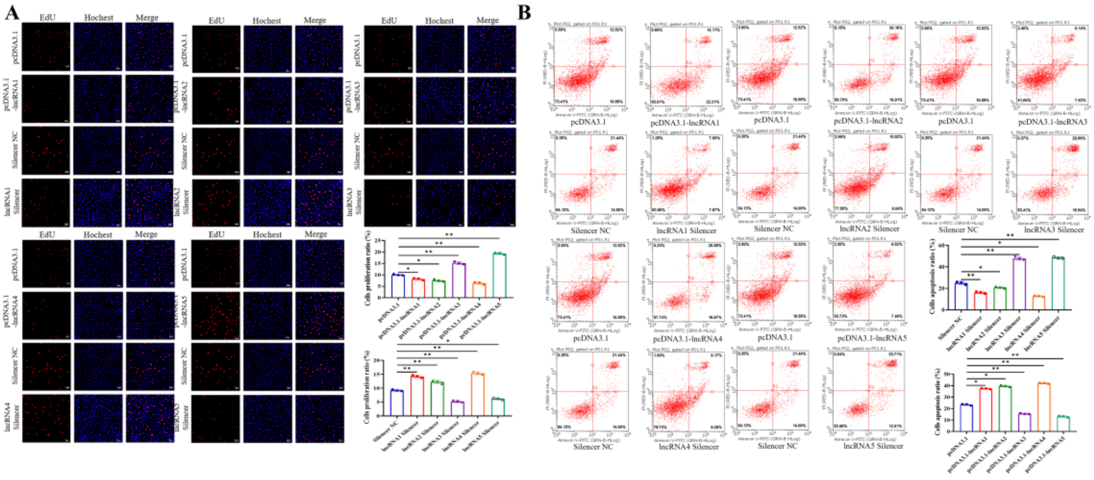


**Fig. SF1. Effects of five lncRNAs on the proliferation (A) and apoptosis (B) of vascular cells.**

| Gene name | Primer sequences (5′ to 3′) | Size (bp) | Accession number |
|---|---|---|---|
| PCNA | F: TCGTTGTGATTCCACCACCAT R: TGTCTTCATTGCCAGCACATTT | 278 | NM_001291925.1 |
| BCL2 | F: GATGCCTTTGTGGAGCTGTATG R: CCCGTGGACTTCACTTATGG | 145 | XM_021099593.1 |
| MCL1 | F: GAAGGCGTTAGAGACCCTGC R: TGCCCCAGTTTGTTACTCCG | 167 | NM_001348806.1 |
| p65 | F: AAGATCTGCCGAGTGAACCG R: GCCTGGTCCCGTGAAATACA | 114 | NM_001404662.1 |
| IkBa | F: AAGTGATCCGCCAGGTGAAG R: CTGCTCACAGGCAAGGTGTA | 188 | NM_020529.3 |
| STAR | F: CGACGTTTAAGCTGTGTGCT R: ATCCATGACCCTGAGGTTGGA | 136 | NM_213755.2 |
| CCNH | F: TGCTGAGCTTGCACTTAACG R: CAGGCTCCTCAGAGTACTGG | 113 | NM_001364075.2 |
| CCNE | F: AGCCTGTGAAAACCCCTGTT R: TCCAGAAGAATCGCTCGCAT | 252 | XM_005653265.2 |
| CDK4 | F: CAGTGTACAAGGCCCGTGAT R: CAGTCGCCTCAGTAAAGCCA | 134 | NM_000075.4 |
| MYC | F:CGTCCTCGGATTCTCTGCTC R:GCTGGTGCATTTTCGGTTGT | 380 | NM_001354870.1 |
| CDK7 | F: ACTTTGGGCACACCAACTGA R: TCTTGGCAGCTGACATCCAG | 246 | NM_001324072.1 |
| CDK2 | F: AAAAGATCGGAGAGGGCACG R: GCAGTACTGGGTACACCCTC | 121 | NM_001285465.1 |
| CCND1 | F: CTTCCATGCGGAAGATCGTG R: TGGAGTTGTCGGTGTAGATGC | 234 | XM_021082686.1 |
| CCNE2 | F: GGGGGATCAGTCCTTGCATT R: AGCCAAACATCCTGTGAGCA | 154 | NM_001243931.1 |
| CDKN1B | F: GGCAAGTACGAGTGGCAAGA R: CGTGTCCTCAGAGTTAGCCG | 171 | NM_004064.5 |
| ATG7 | F: AGCTGGTCATCAATGCTGCT R: AAAAAGCGATGAGCCCAGGA | 143 | NM_001349238.2 |
| ATG5 | F: TGCAGATGGACAGTTGCACA R: CCACTGCAGAGGTGTTTCCA | 139 | NM_001286106.2 |
| LC3 | F: TGGGCTCAAGCAATTCTCCA R: AAATCCCGGTGATCATCGAG | 290 | XM_011529084.3 |
| p62 | F: TTCCTCTCGCTGTCCTGGTA R: GAGTCAGTCTCGGCGATCAG | 212 | NM_014275.5 |
| mTOR | F: TGCAGAGACTTGATGGAGGAG R: TTCAAAGCTGCCAAGCGTTC | 287 | NM_001386501.1 |
| CASP3 | F: ACATGGAAGCAAATCAATGGAC R: TGCAGCATCCACATCTGTAC | 154 | NM_214131.1 |
| CASP8 | F: GAGCCTGGACTACATCCCAC R: GTCCTTCAATTCCGACCTGG | 283 | NM_001031779.2 |
| CASP9 | F: GCTGAACCGTGAGCTTTTCA R: CCTGGCCTGTGTCCTCTAAG | 161 | XM_003127618.4 |
| GAPDH | F: TCGGAGTGAACGGATTTGGC R: TGACAAGCTTCCCGTTCTCC | 189 | NM_001206359.1 |

**Table S1. The information for the highly differentially expressed lncRNAs that we identified, related to Figure 1.**

| test_id | TCONS_00248283 | TCONS_00102237 | TCONS_00095111 | TCONS_00226099 | TCONS_00150976 |
|---|---|---|---|---|---|
| gene_id | XLOC_110286 | XLOC_025806 | XLOC_075168 | XLOC_102237 | XLOC_057528 |
| lncRNA_locus | chr15:43295800-43299401 | chr1:176991246-176991613 | chr13:73518992-73519539 | chr15:44838886-44850522 | chr12:55983431-55984160 |
| status | OK | OK | OK | OK | OK |
| JMa_fpkm | 5.49595 | 1.95021 | 0.708047 | 0.514824 | 0.221397 |
| JMb_fpkm | 55.326 | 4.39335 | 1.58925 | 1.09253 | 0.740667 |
| log2(fold_change) | -3.33152 | -1.17169 | -1.16643 | -1.08552 | -1.74219 |
| regulation | down | down | down | down | down |
| p_value | 0.00005 | 0.02855 | 0.0492 | 0.03765 | 0.0451 |
| significant | yes | yes | yes | yes | yes |
| related_gene | ANGPT2 | BCL2 | ATG7 | VEGFC | TP53 |
| gene_locus | chr15:42687923-42787653 | chr1:175824218-175994403 | chr13:74636936-74915764 | chr15:44742834-44773339 | chr12:55220545-55234668 |
| gene_exon | 9 | 4 | 29 | 8 | 11 |
| gene_mRNA | （1..7323，34974..35129，38198..38319，40637..40869，42811..42938，47615..47716，48280..48446，52224..52354，55575..56749） | (1..1924, 85471..86668, 176864..180812) | （1..119，136067..136193，136386..136558，137292..137346，145373..145490，147262..147339，150855..150971，152529..152678，167883..167971，169458..169591，174641..174731，175502..175646，182083..182241，187731..187925，188424..188627，190320..190435，199775..199850，215806..215886，279379..279501，395023..397385） | (1..3445, 66441..66654, 68378..68568, 78440..78596) | （1..112, 9385..9486, 9623..9644, 9730..9984, 10427..10613, 10703..10815, 11039..11148, 11476..11603, 11700..11773, 12639..12745, 13533..14144） |

**Table S2. The information for all qPCR primers used in this study.**
